# Keep Your Opponents Close: Social Context Affects EEG and fEMG Linkage in a Turn-Based Computer Game

**DOI:** 10.1371/journal.pone.0078795

**Published:** 2013-11-20

**Authors:** Michiel M. Spapé, J. Matias Kivikangas, Simo Järvelä, Ilkka Kosunen, Giulio Jacucci, Niklas Ravaja

**Affiliations:** 1 Helsinki Institute for Information Technology, Aalto University, Helsinki, Finland; 2 School of Business, Aalto University, Helsinki, Finland; 3 Department of Computer Science, University of Helsinki, Helsinki, Finland; 4 Department of Social Research, University of Helsinki, Helsinki, Finland; University of Tuebingen Medical School, Germany

## Abstract

In daily life, we often copy the gestures and expressions of those we communicate with, but recent evidence shows that such mimicry has a physiological counterpart: interaction elicits linkage, which is a concordance between the biological signals of those involved. To find out how the type of social interaction affects linkage, pairs of participants played a turn-based computer game in which the level of competition was systematically varied between cooperation and competition. Linkage in the beta and gamma frequency bands was observed in the EEG, especially when the participants played directly against each other. Emotional expression, measured using facial EMG, reflected this pattern, with the most competitive condition showing enhanced linkage over the facial muscle-regions involved in smiling. These effects were found to be related to self-reported social presence: linkage in positive emotional expression was associated with self-reported shared negative feelings. The observed effects confirmed the hypothesis that the social context affected the degree to which participants had similar reactions to their environment and consequently showed similar patterns of brain activity. We discuss the functional resemblance between linkage, as an indicator of a shared physiology and affect, and the well-known mirror neuron system, and how they relate to social functions like empathy.

## Introduction

This study concerns the degree to which people who communicate share more than just words. We copy emotions and gestures from those we communicate with, but recent studies indicate that even our nervous systems show evidence of reflection [1], [2]. The present study investigated the degree to which the social context affects physiological linkage.

### Imitation and social interaction

Psychologists have long maintained that communication and imitation require a mental representation – or “model” – of the other, which links observed actions to one's own behaviour [3], [4]. A recent example of this idea can be found in the mirror neuron system (MNS, [5]), a hypothesised mechanism based on the finding that groups of neurons in the premotor cortex of macaques fired both when the monkeys performed an action and when they observed the same action in others [6]. Thus, a fundamental neuronal similarity between the acting self and the observed other is established which is believed to provide a source of shared representations that enables understanding perceived actions and imitating others (but see [7]). Furthermore, the idea of a common coding between the self and the other inspired continued research with evidence for the involvement of the MNS in a variety of complex social functions, such as language production [8], having a theory of mind [9], showing empathy [10], [11] and exhibiting social understanding [12].

### Two-person neuroscience of linkage

The way we internalise the behaviour of others, however, goes well beyond the representation of the other: our very physiology shows a task-related commonality. Thus, people do not only mimic one another in their gestures and facial expressions, but even their brain- and body signals copy one another. The coordination of psychophysiological changes in two persons has been referred to as “physiological linkage” (e.g., [13]) and “physiological compliance” [14]. We will refer to this effect, throughout the present article, as *linkage* in order to differentiate it from associated behaviour (e.g., mimicry, imitation, communication), measurements (coherence, connectivity), and terms that presuppose an interpretation (compliance, contagion).

Recent advances in neuroscience have made it possible to record the physiological signals of multiple persons simultaneously, paving the way for a two-person neuroscience [15] and allowing researchers to study linkage in a variety of scenarios. In general, the effect is associated with periods of intense social interaction [16], such as during marital conflict and psychotherapy sessions [13], [17], [18]. Evidence of linkage was also found between mothers and their 3-month old infants, whose heart-rates show a coordinated rhythm during face-to-face interactions [1]. Likewise, adults performing in a fire-walking ritual show synchronized arousal in terms of heart-rate with their audience [19]. Adding facial electromyography (fEMG) and electrodermal activity measurements, Chanel, Kivikangas and Ravaja [20] found that indices of linkage were related to the degree to which a computer-game, played by pairs of participants (dyads), was competitive.

There are various explanations for the emergence of physiological linkage, but shared or communicated *emotions* (i.e., emotion contagion) are consistently found to be pivotal [21], without necessarily addressing whether they are the cause or result of linkage. The first studies on linkage reported an association with strong negative affect [13], [17], but positive emotions have also been reported [18]. The emotional reflection may thus underlie the generation of a “shared affective space” similar to the workings of the MNS, with brain regions following congruent patterns of activations between the interacting partners [22]. Similarly, it has been found that watching emotional events creates a type of “cortical consensus”: brain regions are activated synchronously across subjects due to the emotional trigger [23]. Moreover, there is evidence that emotional proximity between an interviewer and his/her patient predicts both perceived empathy of the interviewer, and their common physiological linkage [24]. Chanel et al. [20] also showed that linkage during digital game playing was associated with self-reported “social presence” – a sense of “being together” – including interpersonal understanding and attention.

However, a significant drawback of the studies discussed thus far is the difficulty in disentangling cause and effect concerning the relationships between mimicry/imitation, physiological linkage, social effects and emotion. For example, Hove and Risen [25] showed that finger-tapping-synchrony between participants and the experimenter predicted affiliation with the experimenter, possibly because observing mimicry itself may trigger the reward system and result in positive emotions [26]. Thus, mimicry itself causes emotional changes, making it unclear whether linkage in the absence of synchronised action even exists, or whether linkage is a mere epiphenomenon to synchronous action. Since studies – such as [20], in which participants played a computer game simultaneously – typically do not explicitly control for synchronous action, it is possible that the behavioural co-ordination confounded both linkage and experience.

### Present study and hypotheses

In the present study, we set out to discover whether linkage could be observed in the absence of synchronised action. Inspired by the finding of Chanel et al. [20] that a competitive social context may increase linkage, the present experiment utilised a design in which the level of competition within a digital computer-game was systematically varied. Crucially, unlike similar studies (such as [20]), the game was played in a strictly turn-based manner. In other words, only one participant was behaviourally interacting with the game at any given time, whilst the other participant could only observe the consequences of the former's actions. Thus, we ensured that the linkage could be studied independently from simultaneous, manual action, or behavioural concordance.

In order to learn how the type of interaction between participants influenced their linkage, we manipulated the level of competitiveness during the four games. In the most cooperative condition (C1), the two players took alternating turns to command a single team of 6 game characters, with no distinction between the two players, against a single computer-controlled team. In the second-most cooperative condition (C2), participants controlled separate teams of 3 game characters each, but were allied against two computer-controlled teams, and were asked to compete for being more effective against the computer-controlled team. In a yet more competitive condition (C3), the two players likewise commanded separate teams of 3 game characters, but played *against* one another and were partnered with distinct computer-controlled teams (i.e., the setting mirrored that of the previous one, but with one computer-controlled and one player's team exchanged with each other). Finally, in the most competitive condition (C4), there were no computer-controlled teams at all, and players were pitted directly against each other.

To test 1) whether physiological linkage would occur in the absence of co-occurring action (in a game where the players play and watch the other one play alternately) and 2) whether linkage would increase as a function of competition (as reported by [20]), we measured types of interpersonal synchrony both in electroencephalography (EEG) and fEMG. Linkage in fEMG was expected to provide information on the degree to which facial expressions and emotions are shared between participants. EEG measurements of connectivity were expected to inform us as to whether a type of linkage as in [2] could be observed, but in relation to the type of match, rather than to specific, pre-determined events that took place during the match. Further, this made it possible to study whether social effects similarly affect EEG linkage, and thereby provide important information on the hypothesised relationship between social context, affect, and linkage.

Since EEG activity in different frequency bands is traditionally associated with distinct functions, task-related power effects were additionally studied. In the study [2], for instance, phase synchronisation effects between the pairs of guitarists seem to mainly occur at theta (peaking at 4.95 Hz) frequency, which they suggest may be indicative of the voluntary motor control aspect involved in interpersonally coordinating movement. They further note how such interpersonal neuronal oscillations may be of critical importance of social organisation and ‘theory of mind’ capabilities. Yet, these functions, in the hypothesised link between autism and the mirror neuron system, are more often associated with oscillations in the “mu” frequency [27] - generally placed somewhere between the alpha (8–12 Hz), and lower beta (13–18 Hz) frequencies. Given the lack of consensus on the spectral properties of EEG linkage, we used a full set of classical bins spanning the theta (4–8 Hz), alpha (8–12 Hz), beta (13–29 Hz), gamma (30–45 Hz) and higher-gamma (55–80 Hz) frequencies, and matched observed power effects with interpersonal coherence effects in order to explore whether common patterns could be established.

Finally, in order to better understand the phenomenology behind the observed linkage, participants were asked to complete questionnaires. The social presence module of the Game Experience Questionnaire (SPGQ) was previously found to show significant associations with fEMG linkage ([20], see [28] for an updated version of the SPGQ),. This module includes three scales - social empathy, negative feelings and behavioural involvement – that quantify the degree of social richness felt during the game.

## Materials and Methods

### Participants and ethics statement

Recruitment of participants was conducted by advertising the study over student internet mailing lists, after which forty-one pairs (dyads) of participants volunteered in exchange for cinema tickets. Dyads were self-reported friends and were of same sex, with 25 male and 16 female pairs. Due to technical difficulties, data from 3 (male) dyads were not included in the analysis. In accordance with the declaration of Helsinki, participants were briefed on the purpose and procedure of the study and signed informed consent prior to the experiment. Participants were also reminded that they could withdraw from the study at any time without fearing negative consequences. The study did not concern medical research and, in accordance with Finnish law, the need for formal approval was waived by both the Vice President of Aalto University and by the Chairman of the Ethics Review Board of Aalto.

### Apparatus and stimulus material

**Figure 1:**
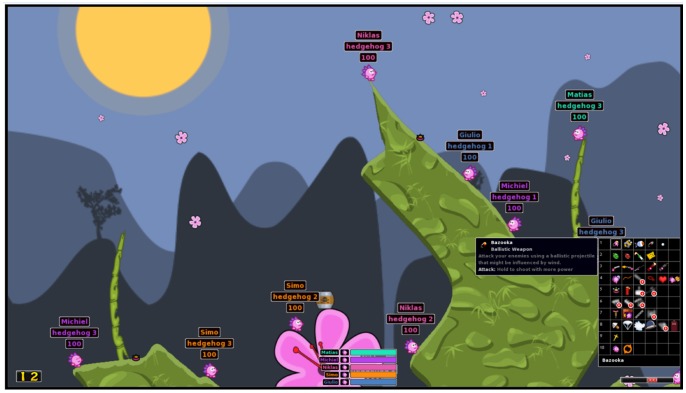
A screen-capture of Hedgewars. Participants commanded teams of hedgehogs and engaged in ballistic shooting (weapon choices are portrayed in the menu on the right) with or against other players.

Participants engaged in the turn-based artillery game of *Hedgewars* (see figure 1; http://hedgewars.org), an open-source version of the more commonly known artillery game of *Worms* (Team17 Software Limited, Ossett, UK). In the game, players command teams of game characters positioned on a 2-dimensional landscape and engage in ballistic shooting. The goal of the game is to be the last, remaining team. In order to achieve this, they can use their turn to move the game characters, choose and use a variety of weaponry to destroy the opposing team.

The experiment was run on a Kubuntu 11.04 Linux desktop computer and projected to a 150×110 cm white screen using a Hitachi CP-X328 LCD video projector at a resolution of 1024×768 pixels. Participants shared a single mouse and keyboard to control their team and were seated next to each other at a distance of 1.7 m from the screen.

### Procedure

Prior to the experiment, participants filled out background questionnaires. While the recording equipment was set up (e.g. attaching electrodes), participants practised the game. Immediately prior to the beginning of the experiment, a 5 minute baseline recording took place. Participants then played four matches of Hedgewars, with the order of conditions randomised. Within every game, the team turn order was randomised. Turns had a maximal duration of 45 s, but were ended immediately after firing a weapon. Each match took on average 573.90±231.82 s in total and the entire experiment, including setting up the recording equipment, instructing the participants and filling out the forms, took approximately 2.5 hours for each dyad. Upon completion of a single match, participants filled in a modified version of the social presence module of the SPGQ [28], which is a Likert-scale self-report questionnaire that provides three measures of importance to the social experience in game-play. The scale of Psychological Involvement provided a measure of *empathy* with items such as “I felt connected to the other” and “I found it enjoyable to be with the other”. Psychological Involvement was used as an indicator of *negative feelings* with items like “I felt revengeful” and “I felt *schadenfreude*”. Finally, the scale of *behavioural involvement* provided a measure of the degree to which the actions (as opposed to the emotions) of the other had an impact on their own actions and vice versa, with items such as “What the other did affected what I did” and “I paid close attention to the other”. In order to find out if any specific effect was related to the average experience level of a dyad, we also asked participants to rate their history of playing the more famous Worms game on an ordinal scale ranging from 1 (never played the game) to 5 (played it very often: I have mastered even the more obscure parts of the game). However, as this variable was not significantly correlated with any task-related effect on linkage, we omitted it from further analysis.

### Electrophysiological recording and pre-processing

EEG data were recorded at 256 Hz using two Varioport-B portable bio-signal recorders (Becker Meditec, Karlsruhe, Germany), with six Ag/AgCl electrodes placed on the scalpel (F3/F4/C3/C4/P3/P4), bilaterally referenced using two electrodes clipped to the left and right earlobe. Peri-ocular EEG was recorded at 128 Hz from two sites lateral, inferior and superior to the right eye, combined to form horizontal and vertical electrooculographic (EOG) channels. Finally, facial electromyographic (fEMG) data were recorded at 1024 Hz over sites overlying the left zygomaticus major (ZM), orbicularis oculi (OO) and corrugators supercilii (CS) muscle regions as recommended by [29]. Notice that the first two areas have been related to the occurrence of spontaneous smiles of enjoyment – the so-called *Duchenne* smiles [30]. All physiological data was subsequently resampled using spline interpolation to a common samplerate of 256 Hz.

Data analysis in EEGLAB [31] and Mathworks Matlab involved band-pass filtering the EEG data between 0.5 Hz and 80 Hz, with a notch-filter between 45 and 55 Hz. The data from the two players was combined to a single, dyad-level dataset and divided into five trials, with four conditions (i.e. matches), and one consisting of resting state measurement (240 s). Trials were segmented into epochs of 1000 ms each, with 50% overlap, resulting in an average of 1151.34±317.17 epochs, after which an automatic artifact correction, based on the EOG channels, was applied [32]. Following, epochs with suspect activity were removed using the threshold-based artifact rejection algorithms in EEGLAB.

### Analysis

Power spectra were obtained by first band-pass filtering using a 3rd order Butterworth filter for the specific frequency band, then using fast Fourier transform in EEGLAB to calculate spectra for theta (4–8 Hz), alpha (8–12 Hz), beta (13–29 Hz), gamma (30–45 Hz) and higher gamma (55–80 Hz) bands. Power values were normalised using the natural log transform and averaged within conditions and between the two participants within each dyad. In order to establish both intra- and inter-personal connectivity, we measured classical coherence. Classical coherence is a measure of coupling that quantifies the linear relationship between two signals at a given frequency as a value between 0 (no correlation) and 1 (perfect correlation). Epochs were Hanning-windowed and transformed to time/frequency domain by using the Short Term Fourier Transform. To control for the number of data-points under consideration, separate coherence values were calculated over sets of 199 epochs, with 50% overlap, and averaged across sets within a condition. With 12 channels over two participants, and 5 frequency bands, a full analysis would require 330 different dependents, making the chance of observing spurious significance a near certainty. To avoid this, networks of functional connectivity were established based on a region of interest (ROI) method with the three regions comprising a select number of channel pairs, comprising the frontal (F3-F3, F3-F4, F4-F3, F4-F4), central (C3-C3, C3-C4, C4-C3, C4-C4) and parietal (P3-P3, P3-P4, P4-P3, P4-P4) region. Coherence values for each ROI were normalised using the inverse hyperbolic tangent and averaged across each ROI.

EMG data were high-pass filtered using a Butterworth filter at 5 Hz [33] with initial segmentation into five single trials for each dyad, similar to those of the EEG. After applying full-wave rectification, EMG data was smoothed by using a first-order moving average filter over 33 samples. Based on previous work suggesting that smiles of enjoyment last typically between 0.5 and 4 s [34], zero-lag cross-correlation scores were calculated using windows of 4 s and intervals of 0.5 s The resulting 1006.7±493.9 scores were squared and averaged to create single R^2^ values for each condition.

To test how competition in gameplay affected EMG correlation, EEG inter-personal synchrony and power, a consistent, two-step approach was employed. First, the task-related effect was calculated as the average across the four game-play conditions, and contrasted using paired samples T-test with the baseline. Second, repeated measures ANOVAs were carried out only for those frequency bands showing an effect in the preceding step. *Competition* was used as a factor with the four types of game-play arranged in increasing level of competition along with the baseline. *Site* was used as a secondary within-subject factor, with 3 levels (for each electrode) in the EMG correlation analysis, 6 levels (one for each electrode) in the power analysis, and 3 levels (one for each group of couplings) in the coherence analyses. We then tested the association between linkage in EMG and social presence, by using three linear mixed models with restricted maximum likelihood estimation to predict the three SPGQ scales (scores averaged across dyad members) using EMG scores of linkage. Dyad ID was specified as the subject variable and playing sequence was specified as the repeated variable, and diagonal was specified (on the basis of Schwarz's Bayesian Criterion, BIC) as the covariance structure for the residuals. Condition and EMG linkage indices for the baseline and game periods were specified as fixed effects. Dyads were treated as a random effect and a random intercept was estimated (with scaled identity covariance structure).

Finally, we included an exploration of co-occurring power-fluctuations to find out whether the short, transient coherence effects could be the building blocks of substantial difference in linkage *between* trials (see [35] for a similar proposal). To establish whether such a *tonic* type of linkage was present, the linkage was calculated as the correlation between the averaged power values for each channel and each frequency band of the subjects. This also provided a degree of effect size and demonstrated that significance of EEG measures did not merely arise from the relatively large sample-size under consideration. All analyses conducted in the present article concerned the shared physiology between two people and were thus conducted at the dyadic level.

## Results

### Power

Task-related power values, computed as the frequency-specific power averaged across participants, sites and levels of competition, were contrasted with resting state power. The tests revealed significant increases in power during the task within the theta, t (37) = 5.39, p<.001, beta, t (37) = 6.40, p<.001, gamma, t (37) = 11.90, p<.001 and higher gamma, t (37) = 11.35, p<.001 frequency bands. The power in alpha frequency was lower, though insignificantly so, t (37) = 1.57.

**Figure 2:**
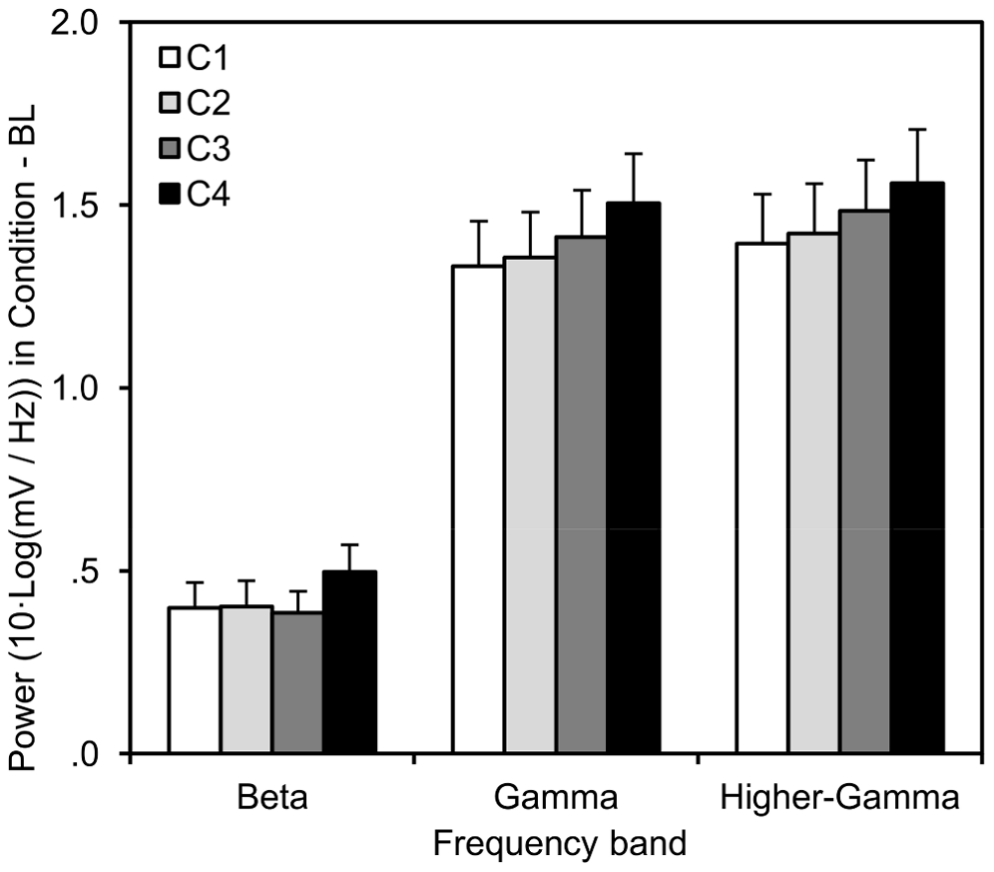
Power effects. Task-related EEG power, averaged over sites, in the beta, gamma and higher gamma frequency bands as a function of competition (C1: cooperative, C2: competitive against computer, C3: competitive with computer, C4: against one another) with baseline (BL) subtracted from each condition. Error bars indicate SE between subjects.

Next, repeated measures ANOVAs showed a significant effect of site on theta, F (5, 33) = 73.20, p<.001, beta, F (5, 33) = 21.47, p<.001, gamma, F (5, 33) = 15.62, p<.001, and higher gamma, F (5, 33) = 14.03, p<.001, generally showing higher power values at frontal electrodes. More importantly, a main effect of competition was not observed for theta power, F (3, 35) = .34, p>.7, but was for beta, F (3, 35) = 3.26, p<.04, gamma, F (3, 35) = 4.74, p<.01, and higher gamma, F (3, 35) = 4.41, p<.02 bands. The interaction between site and competition was neither found for theta, F (15, 23) = 1.67, p>.1, nor beta, F (15, 23) = 1.45, p>.2. However, it was found for both gamma, F (15, 23) = 2.56, p<.03, and higher gamma, F (15, 23) = 2.36, p<.04, bands with greater effects of competition for central and parietal sites as compared to frontal sites. Thus, only in the beta and higher frequency bands were effects of competition observed, as summarised in figure 2.

### Interpersonal coherence

Task-related coherence values were calculated as the frequency-specific coherence averaged across sites and levels of competition and contrasted with resting state coherence. Only the relatively higher frequency bands of beta, t (37) = 2.24, p<.04; gamma, t (37) = 3.49, p<.005 and higher gamma, t (37) = 2.49, p<.02 showed task-related coherence to be higher during the experiment. Neither theta, t (37) = 0.94, p>.3, nor alpha, t (37) = .68, p>.5, showed a significant effect.

**Figure 3:**
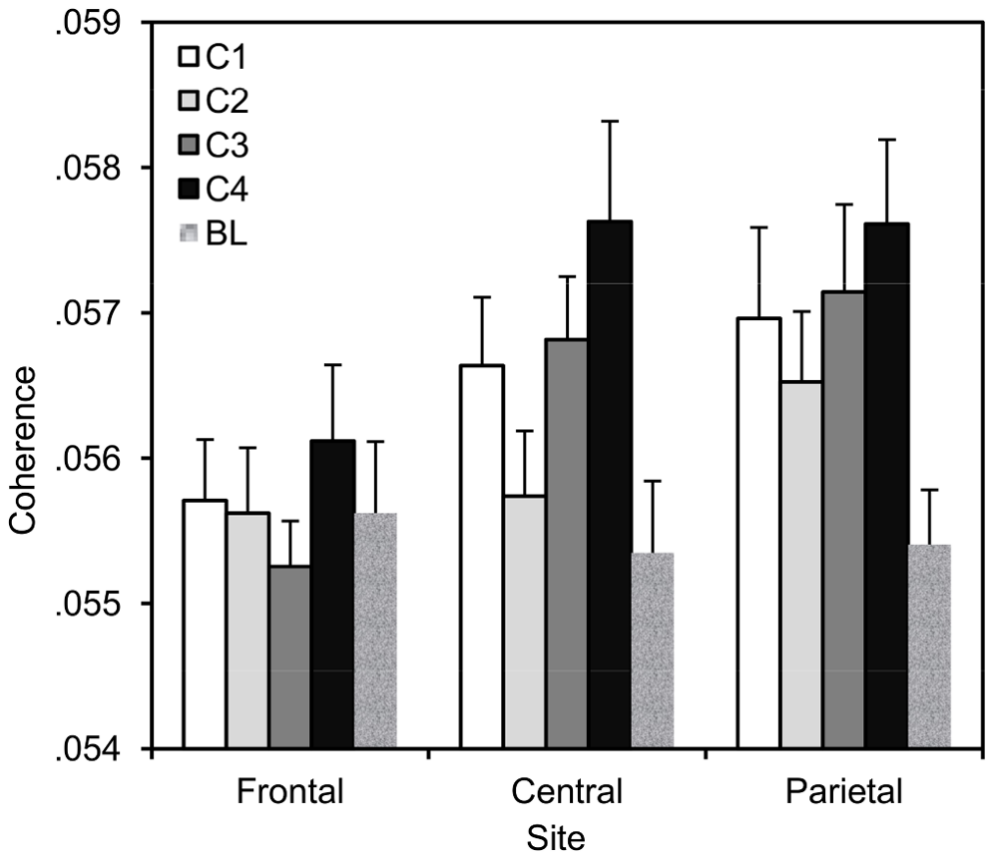
Phasic EEG linkage. Mean interpersonal coherence in beta frequency band as a function of site and competition (C1: cooperative, C2: competitive against computer, C3: competitive with computer, C4: against one another, BL: baseline).

Following, beta band showed a significant effect for site, F(2, 36) = 4.22, p<.03, though the main effect of competition was not significant, F(3, 35) = 2.53, p>.07. However, the interaction between site and competition was significant, F(6,32) = 3.02, p<.02, with the strongest effect of competition occurring over central sites (see figure 3). Tests of linear, quadratic and cubic contrasts showed the effect, in this site, to be best characterised by a linear model term, F(1, 37) = 8.47, p<.007. Neither gamma nor higher gamma band showed significant effects: for site in gamma, F(2, 36) = 2.89 or higher gamma, F(2, 36) = 1.20, competition in gamma F (3, 35) = 1.02 or higher gamma, F(3, 35) = .52, or their interaction in gamma, F (6, 32) = 1.49, or higher gamma (6, 32) = .56. Thus, effects of competition were only observed for the beta frequency.

To reveal the nature of the significant interaction in the beta band, additional repeated measures ANOVAs were carried out for each site. This showed that the effect of competitiveness was significant between central areas, F(3, 35) = 5.07, p = .005, but not between frontal (p>.4) or parietal (p>.16) areas. Pair-wise comparisons showed increased inter-personal synchrony for the two more competitive conditions as compared to the two less competitive conditions.

### EMG correlation

**Figure 4:**
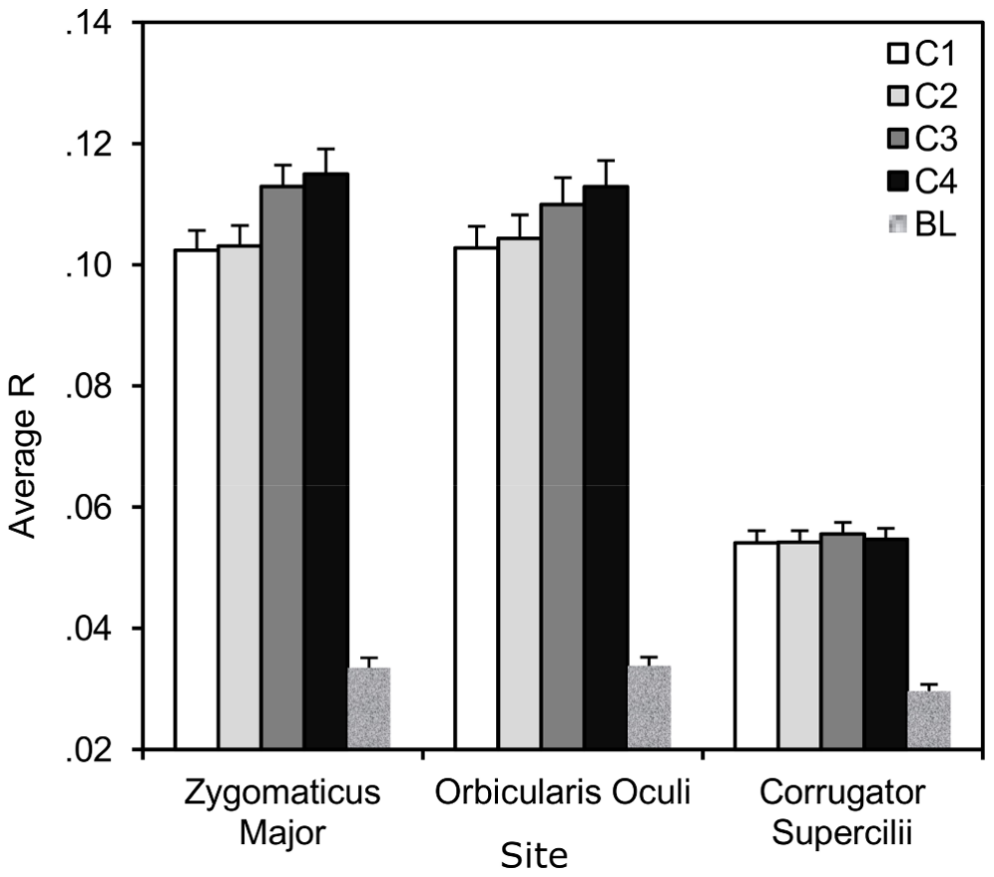
EMG linkage. Mean squared cross-correlation in EMG collected from three facial muscle groups as a function of competition (C1: cooperative, C2: competitive against computer, C3: competitive with computer, C4: against one another, BL: baseline).

Contrasts between the averaged cross-correlation in EMG in tasks versus resting state showed linkage to be present across sites: for sites over ZM, t (37) = 23.51, p<.001, CS, t (37) = 16.87, p<.001, as well as OO, t (37) = 23.54, p<.001, facial muscle groups. A repeated measures ANOVA with site and level of competition as factors showed the observed amount of linkage to depend on the site, F (2, 36) = 154.81, p<.001 and competition, F (3, 35) = 6.08, p<.005. Furthermore, the interaction between site and competition showed that the effect of competition showed differences between sites, F (6, 32) = 2.40, p<.05. To better understand this effect, three separate repeated measures ANOVAs were carried out. This showed competition to have a significant effect on linkage in ZM, F(3, 35) = 6.51, p<.002 and OO, F(3, 35) = 4.09, p<.02, but not in CS, F(3, 35) = 0.66, p>.5. For the two significant sites, higher levels of competition were associated with increased linkage (see figure 4).

In order to explore the similarity between EEG and EMG linkage with regards to the effects of competition, we calculated correlations for the two types of linkage using the task related scores (i.e. the difference between conditions and baseline). Given the functional difference in EEG spectral activity, we used separate scores for the frequency bands, resulting in 180 (3 EEG electrodes×3 EMG electrodes×4 conditions×5 frequency bands) correlations. Amongst these, only 3 had a significance level below p = .01. The best predictor of linkage was found in the COOP condition between the beta frequency coherence in the parietal electrode and both ZM, r = .48, p = .002, and OO, r = .46, p = .004 sites. Furthermore, EMG linkage of OO activity in the VS condition was best predicted by beta frequency coherence over the frontal electrodes.

### Experience questionnaire

Linear mixed model analyses with the three GEQ scales as dependents showed *empathy* to be significantly affected by the degree of competition, F (3, 91.99) = 27.31, p<.001, with the two more cooperative conditions (C1 and C2) showing higher scores than other conditions (C3 and C4; see table 1 for an overview). However, no effects of linkage on experience were observed, *p*s>.2. For *behavioural involvement*, an effect of condition was likewise found, F (3, 94.60) = 16.70, p<.001, with higher scores in the most cooperative condition than in competitive versus computer team, versus one another without and with another human player in the team. Again, linkage did not significantly predict experience on this scale either, *p*s>.15. *Negative feelings*, however, was significantly predicted by and positively associated with linkage in ZM, F (1, 109.54) = 5.l21, p<.03, as well as OO, F (1, 116.57) = 15.83, p<.001, and showed a significant effect of condition, F (3, 83.70) = 4.99, p<.005. Higher scores in this scale were observed for the condition in which participants played directly against one another, rather than together against the computer or in the C2 and C3 conditions. In all models, the random effect of dyad was significant, Wald Zs>3.05, ps<.003.

**Table 1:** Condition and EMG Linkage as Predictors of Social Presence.

	Mean level				*p*			
Scale	C1	C2	C3	C4	Condition	ZM	CS	OO
Empathy	3.92 (.09)	3.72 (.08)	3.34 (.09)	3.26 (.09)	***			
Involvement	4.12 (.10)	3.81 (.09)	3.42 (.10)	3.88 (.10)	***			
Negative feelings	2.86 (.08)	2.90 (.08)	2.90 (.08)	3.09 (.08)	**	*		***

*Note.* Listed are means and SEs for the four conditions (C1: cooperative, C2: competitive against computer, C3: competitive with computer, C4: against one another, BL: baseline) and the significance for the predictors Condition and linkage, over zygomaticus major (ZM), corrugator supercilii (CS), and orbicularis oculi (OO) muscle regions.

* p<.05,

** p<.01,

*** p<.001.

### Power correlation

**Figure 5:**
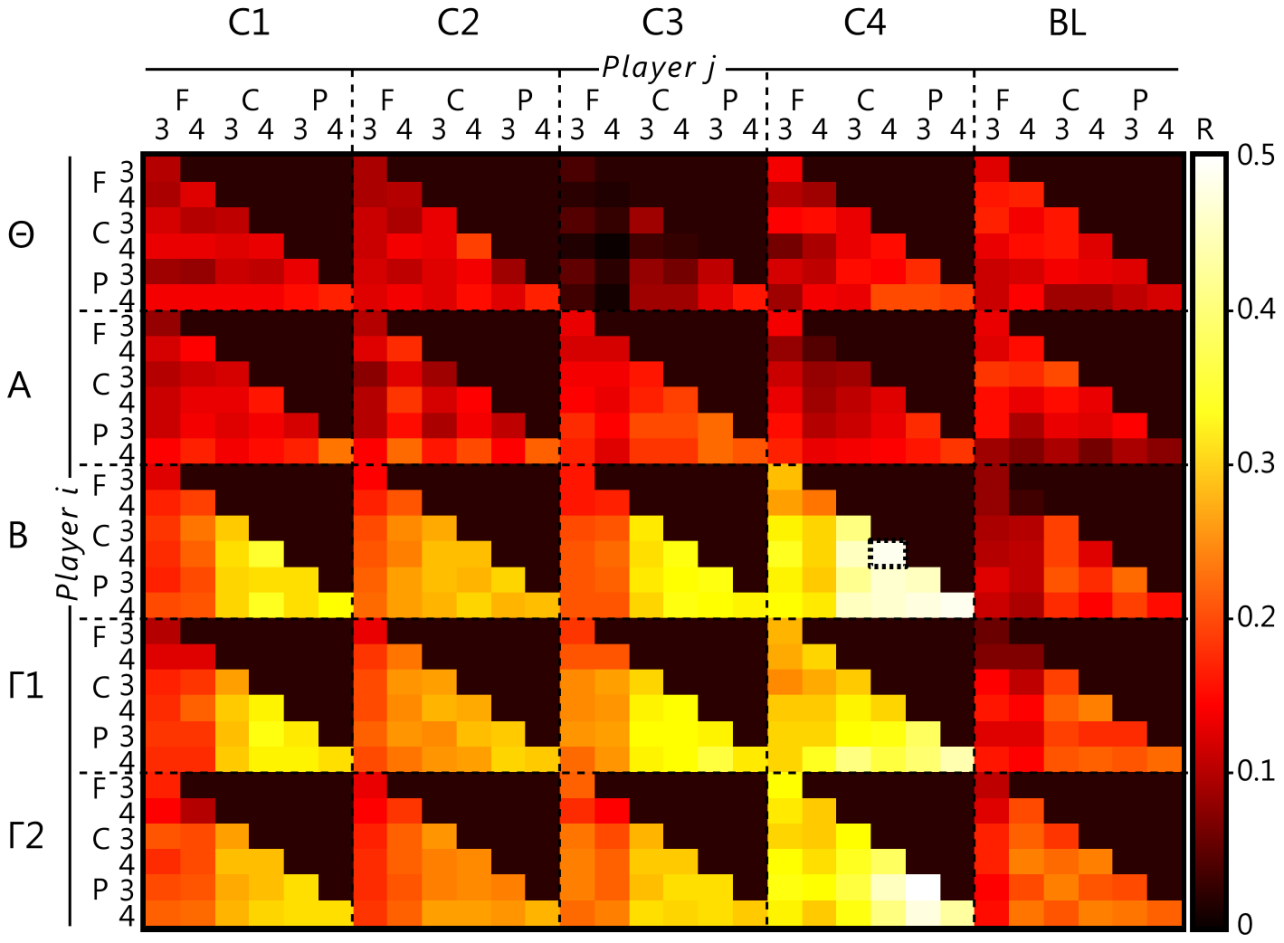
Tonic EEG linkage. Correlation of power between epochs in five frequency bands (theta, Θ: 4–8 Hz, alpha, A: 8–12 Hz, beta, B: 13–28 Hz, gamma, Γ1: 30–45 Hz and higher gamma, Γ2: 55–80 Hz) between the EEG of two participants over six electrodes (F3/4, C3/4, P3/4) as a function of competition (C1: cooperative, C2: competitive against computer, C3: competitive with computer, C4: against one another, BL: baseline). As an example, the dotted line surrounding a single cell (also indicating the highest difference compared to baseline), occurs in condition C4, beta frequency band, between the right central electrode (C4) of player_i_ and of player_j_.

An exploratory analysis was carried out to establish whether power fluctuations correlated between dyads, and if such correlations were affected by the competition factor. Figure 5 illustrates that global effects of condition-dependent increases in tonic linkage can indeed be readily observed in the correlated EEG power of the players, with Variance Accounted For being less than 1% (R = .10) in baseline and the least competitive conditions, but up to ca. 25% (R = .50) in the higher frequency values of centro-parietal electrodes within the most competitive conditions.

## Discussion

We studied the effects of social context on linkage, as measured in interpersonal EEG synchrony of fluctuations in EEG power and facial EMG activity when co-located interactants were playing a turn-based digital game. Increased beta and gamma EEG power was observed, particularly over central and parietal sites, for the more competitive conditions. Similar effects, in terms of location and spectral identity, were found also to occur *between* people, with interpersonal coherence and cross-correlations in power fluctuations increasing along with the degree of competition. Finally, the fEMG results show a clear pattern of positive, emotive linkage: only in those sites associated with the occurrence of *Duchenne smiles* [30] was a strong effect of competition on linkage observed. Condition-specific linkage in EEG partially predicted fEMG linkage. Both the sites and the frequencies provide important indications as to what could be the cause of the linkage. The mirror neuron system, in its reliance on the motor system in general, has often been localised as encompassing at least part of the premotor areas of the human cortex [36]. In terms of EEG, studies have generally related the mirror neuron system to motor-related sites and frequencies, such as with mu-blocking [27], [37] and phi-complex rhythms [38] that tend to occur in the higher alpha and lower beta spectrum of centro-parietal sites.

The beta frequency activity is often associated with motor activity, in the sense that suppression of beta activity has been found to occur both during motor preparation and execution. However, in the present study, beta power *increases* were found to be associated with a degree of social competition. An intriguing explanation for this pattern would be that the beta oscillations – which have been related to functions of motor control [39] and inhibitory mechanisms [40] – resulted from an increased need to suppress imitative or engaged behaviour. Given the localisation of EEG linkage as mainly occurring in the electrodes overlying the motor cortex, the present study provides another indication of the role of the motor system in maintaining a model of the other person. Indeed, given that our results show concurrent affect being associated with increased linkage, the overall picture resembles what is known about the hypothetical relationship between imitation and the mirror neuron system on the one hand and theory of mind and empathy on the other, as briefly discussed in the introduction [11].

Changes in gamma-band activity – although not as clearly present through the various methods in the present study – may also fit this tentative pattern. One could argue that some of the hypothesised functions that have been related to the gamma band, such as binding and conscious awareness [41], [42], are not an incredible leap of imagination away from respectively social linkage and attending another person's action outcomes. This might inspire the idea that linkage is a mechanism of social binding, bringing the consciousness of two persons closer together. However, there are two obstacles that make such hypothesizing premature. First, the academic community is far from reaching a consensus on the functional properties and confounds of gamma oscillations [43]. Second, and this holds similarly true for the beta linkage observed in the present study, even if networks of certain frequencies within one cortex are related to a certain function, this does not necessarily imply that the commonalities *between subjects* play out over the same frequencies. The present study, however, represents one of the first studies in which longer-term linkage between EEG oscillations is observed and we hope the spectral-specific aspect and its relation to social context can be addressed in future research.

Overall, the results strongly support the hypothesis of the social context – operationalised here as the degree of competition – affecting the linkage between two people across a range of indices. Given the degree to which fEMG correlation was affected by this relationship, we propose it is likely that the emotional, co-affective aspect is a crucial aspect of linkage.

The present study partly replicates findings that showed competition affects linkage [20]. They used a similar setup of dyads playing a computer game in cooperative and competitive modes and recorded facial EMG, as well as ECG, respiration rate and electro-dermal activity. We replicated their observations of increased linkage in peripheral physiology and the association with a degree of social presence (but only in terms of negative feelings). Extending their findings, we provide evidence of similar patterns of linkage occurring in central, psychophysiological signals, such as EEG. Moreover, we show that the simultaneous behaviour is not a requirement for linkage to occur: unlike [20], our participants were engaged in a turn-based game and were thus not simultaneously, behaviourally engaged, which could be a critical confound for linkage.

The finding that more linkage is observed if two persons play *against* rather than *with* each other may strike one as rather counter-intuitive. However, it is possible that increased linkage during competition could fulfil an adaptive strategy. In a competitive, social situation, a player wishing to win may more vigilantly attend the (in-game and out-game) actions of the other player (e.g., to ensure that one will react at least equally well in a corresponding situation). This enhanced vigilance may evoke similar mental models in the players, thereby resulting in increased physiological linkage. Nummenmaa et al. [23] also found that inter-subject synchrony of brain activity in the emotion-processing and default-mode networks is greater during cinematic events that were evaluated as being negatively valenced. This is in line with the present finding in that the most competitive condition elicited highest negative feelings (e.g., vengefulness and *schadenfreude*), although, obviously, competition may elicit also positive emotions in the context of games. An additional function for increased linkage in competitive situations could be that linkage may play a compensatory role. That is, reacting in a similar way to the other person (e.g., smiling when the other person smiles) could serve to maintain a friendly relationship in the face of potential dangers associated with competition. Indeed, the manifestation of togetherness, such as linkage provides, may paradoxically be of most importance when a bond may otherwise be severed.

### Limitations

As effective as the present setup was in establishing clear social effects on linkage in a dyadic manner without behavioural synchrony, future research should be aware of the limitations within the present study. One important aspect is that it can be extremely difficult to disentangle behavioural from psychophysiological synchrony. On a theoretical level, it is impossible at this point to conclude either that the mirrored expressions picked up by fEMG *caused*, as opposed to *are caused by*, the changes in interpersonal EEG coherence. It is clear that future research must provide answers to this important question, possibly by employing an event-related approach to independently manipulate common affect and cognition.

The present study, in which more spontaneous oscillations and correlations were studied, suffers from a lack of systematic control that is inherent to the natural setting of our experiment. Much like in other event-unrelated studies on linkage (e.g. [18]), it is difficult to establish what exactly drives the effect. Our social context of competition could result in a type of engagement, which could well be the more important predictor of linkage. What exactly constitutes an event of competition or engagement is difficult to define due to the hierarchical nature of events [44]: is it perhaps when one player succeeds in delivering a fatal blow to the other, or is it more a lingering sense that continues and grows throughout the game? We believe our results, and in particular the emerging pattern of convergence between effects of social context on cortical and facial activity, show important pointers to answering these questions.

Furthermore, it is possible that sources of commonality in the measures of coherence and correlation resulted from the dyads of participants sharing a space, and thus the same auditory and visual stimuli, even though they played the game in an alternating manner. One of the likely sources for such an occurrence could in fact be the propagation of fEMG to EEG. However, as EMG contamination from facial muscle activity has been shown to have the strongest effect on frontal gamma activity [45], this seems an unlikely candidate to fully account for the reported data greatest effects on central beta. Common eye-movement activity, resulting from the two participants looking at similar stimuli, would likely have affected frontal activity in frequencies below the alpha band [46] or above the gamma band and thus neither fit the observed pattern. A remaining possibility, of course, is that the social context of the style of gameplay affected implicit behavioural concordance – i.e., the most competitive condition in the game resulting in mirror-like, minute movements and expressions. This, then, is almost indistinguishable from our hypothesis: the social context of gameplay resulted in more similar perception of the environment and more similar affective evaluation thereof.

## Supporting Information

Appendix S1 **Availability of Data.**
(DOC)
