# Supplementary material for: Keep Your Opponents Close: Social Context Affects EEG and fEMG Linkage in a Turn-Based Computer Game
Source: PLoS One. 2013 Nov 20;8(11):e78795. doi: 10.1371/journal.pone.0078795 (PMC3835884; doi:10.1371/journal.pone.0078795)
Supplement: Appendix S1 — Availability of Data. (DOC) [file pone.0078795.s001.doc]

Supporting Information

In accordance with the guidelines of PLOS ONE, the authors state that data for this study can be made available upon request. Please see <http://www.hiit.fi/~sovspape/KYOC_pubdata.zip> (216 MB) for a sample raw data set, synchronized between the members of a single random pair of subjects, including all conditions and baseline. For more information, please see the included file pubdata_README.txt.
